# Early detection of twiddler syndrome due a congestion alert by remote monitoring

**DOI:** 10.1002/ccr3.979

**Published:** 2017-05-04

**Authors:** Satoru Sekimoto, Mai Wakamatsu, Akihiro Morino, Takayuki Yoshida, Tomoaki Saeki, Yoshimasa Murakami

**Affiliations:** ^1^ Division of CardiologyNagoya City East Medical CenterNagoyaJapan; ^2^ Division of Clinical EngineeringNagoya City East Medical CenterNagoyaJapan

**Keywords:** Intrathoracic impedance, remote monitoring, twiddler syndrome

## Abstract

There are often false‐positive alerts of thoracic impedance monitoring; however, the “false‐positive alerts” might indicate any clinical problem of patient. In the present case, an alert for a drop in intrathoracic impedance, which generally indicates exacerbation of heart failure, enabled early detection of twiddler syndrome.

## Introduction

In the era of remote monitoring (RM), it is possible to obtain much information about patients using cardiac implantable electronic devices (CIEDs). RM systems have the potential for early detection of arrhythmia, heart failure, and device failure. This report describes a rare case of twiddler syndrome after implantation of a CIED, which was detected as an alert for intrathoracic impedance as an indicator of heart failure.

## Case Report

An 84‐year‐old woman was diagnosed as having New York Heart Association (NYHA) class 3 chronic heart failure based on the dilated phase of hypertrophic cardiomyopathy and received a defibrillator capable of cardiac resynchronization therapy (CRT‐D) (Unify™CRT‐D, St. JUDE MEDICAL, Saint Paul, MN) for low ejection fraction (EF) (the value of EF was 13%), complete left bundle branch block evident by electrocardiography (QRS duration was 162 msec), and documented nonsustained ventricular tachycardia in May 2012. The generator was implanted in the pocket fashioned in her pectoral subcutaneous space. She also had dementia and had been treated with medication. Her CIED was monitored remotely by the Merlin™.net Patient Care Network (St. JUDE MEDICAL).

**Figure 1:**
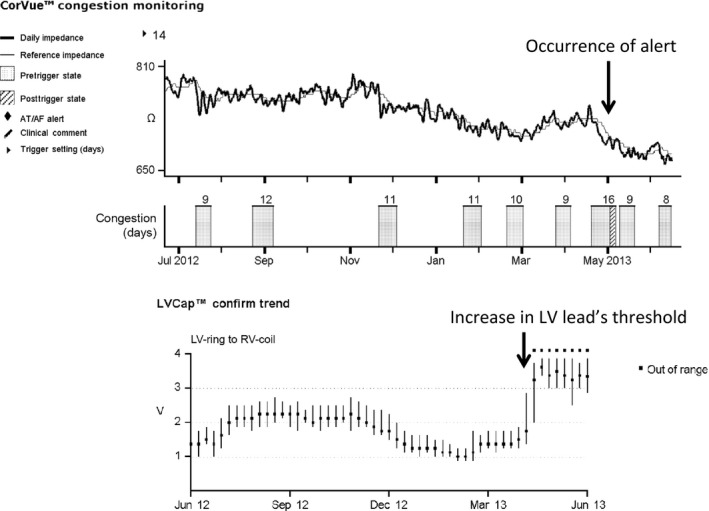
The device data indicated that the intrathoracic impedance began to drop in late April 2013 accompanied by an increase in the left ventricular (LV) lead threshold.

**Figure 2:**
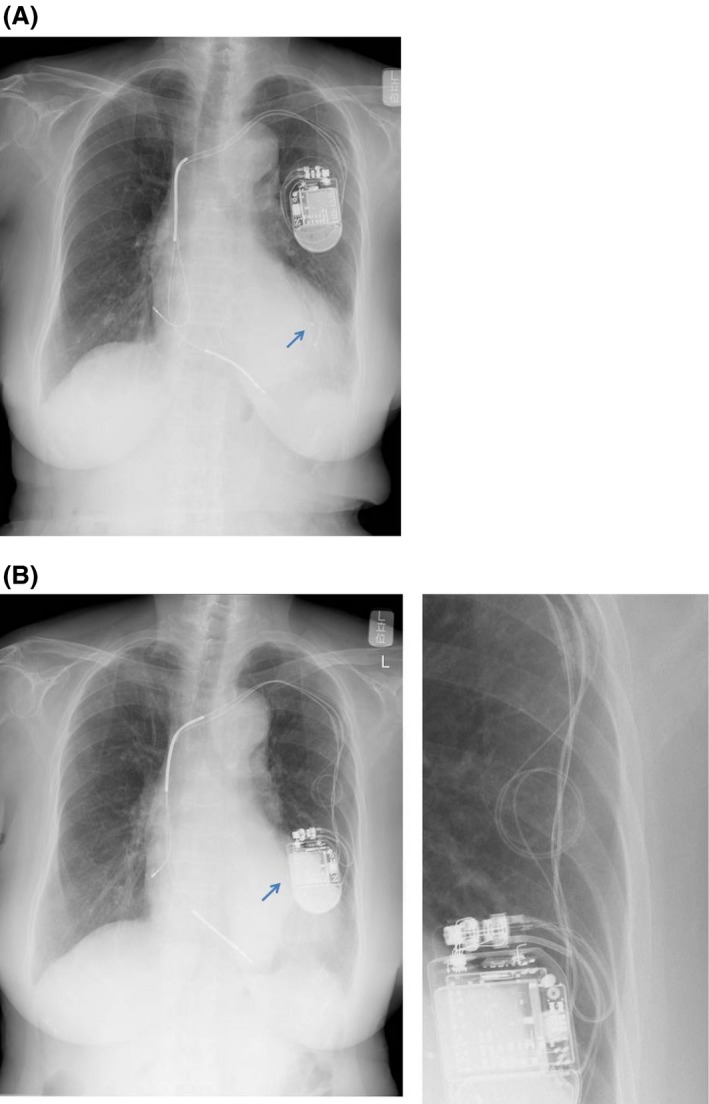
The CIED was located at the correct site just after implantation (A). However, chest radiography 1 year after implantation demonstrated dislodgement of the leads and generator (B). All leads had been pulled. Arrows point the LV lead's tips, which had been pulled proximal direction. Enlarged picture shows twisted leads.

In May 2013, a CorVue™ congestion monitoring alert, associated with a drop in intrathoracic impedance, suggested exacerbation of heart failure. We requested the patient to immediately visit our hospital to check the CIED because of suspected exacerbation of heart failure. The device data indicated that the intrathoracic impedance had dropped in late April 2013 and that the left ventricular (LV) lead threshold had also increased at the same time (Fig. 1). She had no symptoms of heart failure exacerbation such as dyspnea, palpitation, and orthopnea, or physical findings such as edema, weight gain, and a change in the cardiothoracic ratio upon chest radiography. Therefore, we considered that the patient's heart failure had not been exacerbated. Instead, chest radiography revealed twisted leads and generator dislodgement (Fig. 2A and B). In comparison with chest radiography just after implantation, the leads were twisted several fold and the generator had dropped about 12 cm from its position just after implantation. The LV lead had been pulled in the direction of generator dislodgement. We considered that the generator's dislodgement below the chest wall might have induced a decrease in intrathoracic impedance and caused the heart failure alert. The generator could be rotated manually in its pocket and moved in a longitudinal direction. Because of her dementia, the patient had forgotten the presence of the implanted CIED and recognized the generator as a “foreign body” under the skin. This had caused her to manipulate the generator repetitively, resulting in the generator dislodgement and twisted leads.

The patient was hospitalized immediately and underwent surgery for adjustment of the device. The generator's pocket was dilated, and the capsule had become about twice as large as the generator. The generator moved freely in its large capsule, and its leads had become twisted and stretched, as well as having their positions reversed. The data of right atrial (RA) lead and right ventricular (RV) lead were not changed but the threshold of the LV leads was increased to 3.25 V/0.5 msec. Because it was difficult to replace the LV leads, we decided to use the original ones. After the LV lead was inserted as possible into the vein, the threshold was improved to 1.75 V/0.5 msec. The generator was replaced under the greater pectoral muscle and fixed at the periosteum of the clavicle by nylon thread. After the generator replacement, the thoracic impedance had increased by about 50 ohm and recovered to the baseline. The patient was discharged 1 week after, and no generator dislodgement occurred thereafter.

## Discussion

The major findings in this case are that a remote monitoring system enables early detection of twiddler syndrome and the changes in device's parameters were recorded during the occurrence of twiddler syndrome.

Twiddler syndrome, in which twisting or rotating of the device in its pocket results in lead dislodgement and device malfunction, can occur in patients with implanted devices such as a pacemaker, an implantable cardioverter–defibrillator (ICD), cardiac resynchronization therapy (CRT), or a defibrillator allowing cardiac resynchronization therapy (CRT‐D) 1, 2, 3, 4, 5. Deliberate or accidental device rotation leads to lead winding and retraction, causing failure to sense or capture, or stimulation of noncardiac structures including the diaphragm or pectoral muscles. These unfortunate complications of twiddler syndrome have been well described. In most cases, the keys to confirming twiddler syndrome have been loss of capture, noncardiac muscle twitching, or inappropriate ICD discharge. In the present case, a drop in intrathoracic impedance due to misinformation about heart failure by RM enabled us to detect twiddler syndrome.

In the IN‐TIME trial, in which there was a reduction in all‐cause death by automatic multiparameter RM, there were no effects on heart failure‐specific end points including hospital admissions, length of hospital stay, worsening of NYHA class, or worsening of self‐assessed condition 6. Also in randomized data, use of intrathoracic impedance alerts did not improve the outcome of patients with heart failure 7. Furthermore, in the LIMIT‐CHF study, empirical heart failure treatment guided by only intrathoracic impedance alerts did not reduce emergency treatment of heart failure 8. The existence of false‐positive and false‐negative intrathoracic impedance alerts might lead to unfortunate outcomes. Intrathoracic impedance may increase due to emphysema and pneumothorax. Conversely, it may decrease due to causes besides pulmonary congestion, for example, pleural effusion, pneumonia, anemia, and infection of the device. Furthermore, in the present case, shortening of the length between the lead and the generator due to generator dislodgement might have resulted in reduction in intrathoracic impedance. Generally, the electrical resistance is proportional to the length of conductor, so that the reduction in tissue between the lead and the generator might cause the reduction in intrathoracic impedance. Moreover, there were no signs of heart failure exacerbation and no other cause of reduction in intrathoracic impedance. The LV lead impedance showed a simultaneous drop. Traction of the LV lead due to generator dislodgement might result in an increase in the lead's threshold. This concomitant onset of a drop in intrathoracic impedance and an increase in the LV lead threshold increased the possibility that the intrathoracic impedance might have been directly affected by the length between the lead and the generator. Lastly, the recovery of thoracic impedance after relocation also supports this idea.

The cause of twiddler syndrome is an increased pocket size and movement of the generator in the pocket. In this case, the generator was replaced under the greater pectoral muscle and fixed at the periosteum of the clavicle using nylon thread. The implanted limb was strictly fixed by bandaging after reoperation. Thereafter, the generator was well fixed and the twiddler syndrome did not recur.

## Conflict of Interest

No conflict of interests declared.

## Authorship

SS: wrote the initial draft of the manuscript. MW and AM: contributed to analysis and interpretation of data. TY and TS: contributed to critical revision of the article for important intellectual content. YM: involved in final approval of the article.
